# A Simple Model of Optimal Population Coding for Sensory Systems

**DOI:** 10.1371/journal.pcbi.1003761

**Published:** 2014-08-14

**Authors:** Eizaburo Doi, Michael S. Lewicki

**Affiliations:** 1 Electrical Engineering and Computer Science Department, Case Western Reserve University, Cleveland, Ohio, United States of America; University of Tübingen and Max Planck Institute for Biologial Cybernetics, Germany

## Abstract

A fundamental task of a sensory system is to infer information about the environment. It has long been suggested that an important goal of the first stage of this process is to encode the raw sensory signal efficiently by reducing its redundancy in the neural representation. Some redundancy, however, would be expected because it can provide robustness to noise inherent in the system. Encoding the raw sensory signal itself is also problematic, because it contains distortion and noise. The optimal solution would be constrained further by limited biological resources. Here, we analyze a simple theoretical model that incorporates these key aspects of sensory coding, and apply it to conditions in the retina. The model specifies the optimal way to incorporate redundancy in a population of noisy neurons, while also optimally compensating for sensory distortion and noise. Importantly, it allows an arbitrary input-to-output cell ratio between sensory units (photoreceptors) and encoding units (retinal ganglion cells), providing predictions of retinal codes at different eccentricities. Compared to earlier models based on redundancy reduction, the proposed model conveys more information about the original signal. Interestingly, redundancy reduction can be near-optimal when the number of encoding units is limited, such as in the peripheral retina. We show that there exist multiple, equally-optimal solutions whose receptive field structure and organization vary significantly. Among these, the one which maximizes the spatial locality of the computation, but not the sparsity of either synaptic weights or neural responses, is consistent with known basic properties of retinal receptive fields. The model further predicts that receptive field structure changes less with light adaptation at higher input-to-output cell ratios, such as in the periphery.

## Author Summary

Studies of the computational principles of sensory coding have largely focused on the redundancy reduction hypothesis, which posits that a neural population should encode the raw sensory signal efficiently by reducing its redundancy. Models based on this idea, however, have not taken into account some important aspects of sensory systems. First, neurons are noisy, and therefore, some redundancy in the code can be useful for transmitting information reliably. Second, the sensory signal itself is noisy, which should be counteracted as early as possible in the sensory pathway. Finally, neural resources such as the number of neurons are limited, which should strongly affect the form of the sensory code. Here we examine a simple model that takes all these factors into account. We find that the model conveys more information compared to redundancy reduction. When applied to the retina, the model provides a unified functional account for several known properties of retinal coding and makes novel predictions that have yet to be tested experimentally. The generality of the framework allows it to model a wide range of conditions and can be applied to predict optimal sensory coding in other systems.

## Introduction

Barlow's hypothesis of sensory coding posits that neurons should encode sensory information by reducing the high degree of redundancy in the raw sensory signal [1]–[6], and when applied to natural images, it predicts oriented receptive field organizations [7]–[9]. These results qualitatively match response properties of simple-cells in the primary visual cortex [10]–[13], but not those of retinal output neurons (retinal ganglion cells; RGCs) that exhibit a center-surround type receptive field [14]–[16]. The optic nerve poses a far greater bottleneck for the amount of visual information initially available at cone photoreceptors [17], [18], so why does the non-redundant code not match the neural representation in the retina? Alternatively, if the retina does use an optimal code, what is it optimized for?

**Figure 1:**
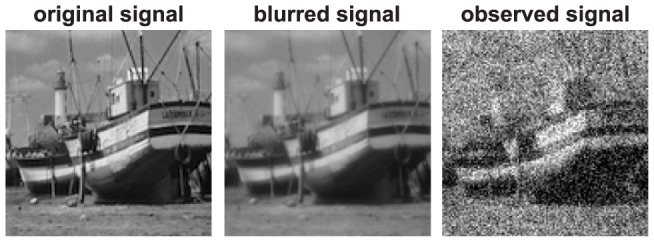
Degradation of sensory signal. Here we illustrate degradation of the image signal in the eye. The *original signal* is a portion of an unaltered standard test image. The *blurred signal* is computed with the blur function measured at 30° eccentricity of the human eye [50]. The *observed signal* (also called the raw sensory signal) simulates the noisy response of cone photoreceptors in a square lattice by adding white gaussian noise to the blurred signal.

Although redundancy reduction has been a guiding principle for understanding sensory coding, there are some important computations and constraints that have not fully been taken into account. The first is that the signal initially available to the sensory system is already degraded, often significantly, and hence forming a non-redundant code of this raw signal does not fully capture the goals of sensory coding. In the retina, for example, the projected image is already degraded by the optics of the eye [19], which is further degraded by photoreceptor noise [20]–[22] (Figure 1). Ideally, those degradations should be counteracted as early as possible in the visual system to avoid representing and processing “noise” in subsequent stages. For this reason, it has been suggested that de-blurring [23], [24] and de-noising [20], [24]–[27] should be important aspects of retinal coding (the latter probably best known by Atick and his colleagues' work).

A second issue is that redundancy reduction does not, by construction, introduce redundancy in a neural population to compensate for neural noise. Neural precision is inherently limited and the information capacity is estimated to be a few bits per spike [18], [28]. Such a limited representational capacity might lead us to hypothesize that individual neurons should represent non-overlapping, independent visual features in order to encode as much information as possible [1], [7], [8]. It has been argued, however, that some redundancy could be useful to convey visual information reliably with noisy neurons [4], [29]–[32], and there is some physiological evidence of redundant codes in neural systems [33]–[36].

**Figure 2:**
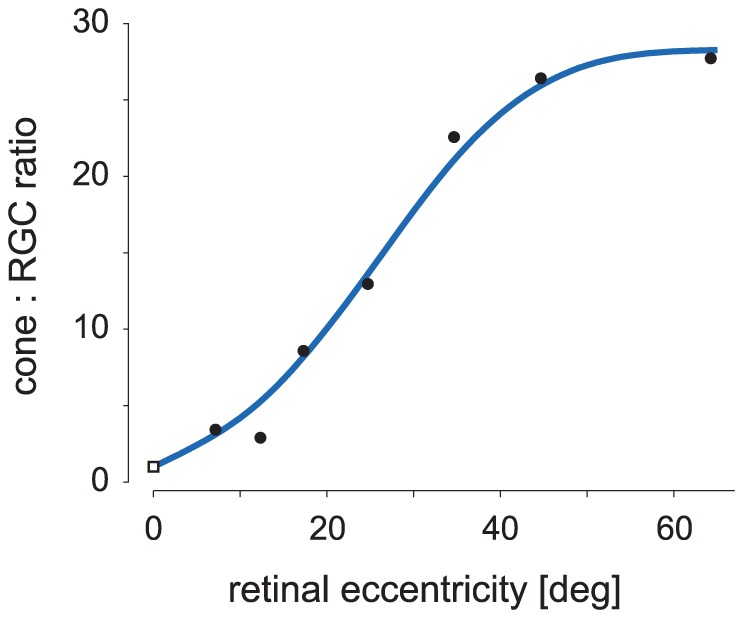
The number of output neurons is far more limited in the peripheral retina. The graph shows the number of cone photoreceptors per midget RGC as a function of eccentricity in the macaque retina. The data at the fovea (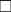) and periphery (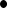) are from [93] and [70], respectively, and the smooth curve was a fit to the data using a cubic spline.

Another issue in predicting optimal codes is that different perceptual systems make different trade-offs to achieve behavioral goals with minimal resources. The most direct way for a system to affect this trade-off in the neural code is to vary the size of the neural population. This, along with the neural precision, determines the total information capacity. In the primate retina this resource constraint is readily apparent. In the fovea, the ratio of cone photoreceptors to RGCs is about 1∶1, but in the periphery the number of RGCs is far more limited – only about 1 RGC for every 25 photoreceptors, for instance (Figure 2). One would expect the optimal neural code to vary significantly across such different conditions, but this issue has not been investigated.

It has also been suggested that resources consumed by neural signaling and connectivity play a role in determining the form of the optimal retinal code [37]–[45]. Any code must extract and transform information from the incoming signal, but there is an inherent cost to doing so, both in terms of the energy to transform and transmit the information and in terms of the physical connections between neurons that subserve the information processing. Energy is always a limited resource, but the physical dimension required for the neural circuits might also be constrained, particularly in the retina where the neural tissue appears to be extremely packed in a highly restricted space. These resource constraints should be balanced against the aforementioned goals of counteracting sensory degradations and forming codes robust to neural noise.

In this article we examine optimal coding of the underlying environmental signal subject to all the aforementioned aspects of sensory systems (signal degradation, neural capacity, and resource constraints) and find that the proposed simple model can account for basic response properties of retinal neurons. Our goal here is to develop a simple model that incorporates key aspects of sensory systems in a unified optimization framework. To achieve this, we make idealizations so that the problem can be analytically well characterized and scales to model large input and output dimensionalities while also accounting for basic properties of sensory systems. In the following, first we systematically contrast the proposed model with a traditional, redundancy reduction model. We find that the optimal model conveys more information about the underlying, original signal, although redundancy reduction can be near-optimal under some conditions. Next, we apply the proposed framework to retinal conditions and find that the concentric center-surround structure of retinal receptive fields can be derived from the optimal model with a constraint of the spatial locality [25], but not with previously examined constraints such as sparse synaptic weights [41] or sparse neural responses [7], [8]. Finally, the proposed model makes a novel prediction that the adaptive change of receptive field structure with different light levels should be much smaller in the periphery than in the fovea due to the much higher cone-to-RGC convergence ratio. An early version of this study was presented as a conference paper [46], and a minimal theoretical analysis of the model was published in [47].

## Results

### The model

**Figure 3:**
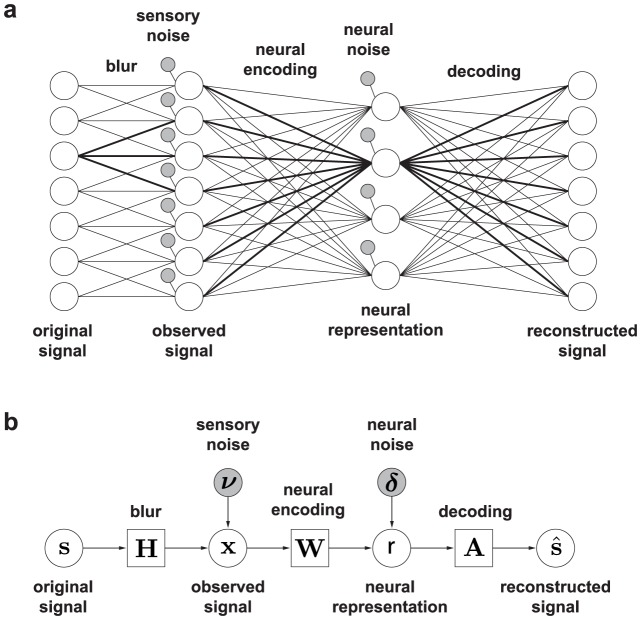
The sensory coding model. (a) Network diagram. Nodes represent individual elements of the indicated variables (noise variables indicated by small gray nodes); lines represent dependencies between them. Bold lines highlight, respectively, a point spread function of the blur from a point in the original signal to the observed signal, an encoding filter (or receptive field) that transforms the observed signal into the neural representation in a single neuron (encoding unit), and a decoding filter (or projective field) which represents the patten of that neuron's contribution in the reconstructed signal (its amplitude is given by the neural representation). In this diagram, the number of coding units at the neural representation is smaller than that of sensory units at the observed signal, which is called an undercomplete representation. Note that the proposed model is general and could form an optimal code with an arbitrary number of neurons, including complete and overcomplete cases. (b) The block flow diagram of the same model using the model variables defined in Methods. Each stage of sensory representation is depicted by a circle; each transformation by a square; each noise by a gray circle.

The proposed model is illustrated in Figure 3. The model forms an optimally robust code in the sense that the original sensory signal can be reconstructed from the neural representation with minimum mean squared error (MSE) despite sensory degradation, neural noise, and a limited number of neurons. The model assumes that the environmental or *original signal* is degraded by *blur* followed by additive noise (*sensory noise*) resulting in the *observed signal*. The *neural representation* is computed with the optimal linear transformation (*neural encoding*) of the observed signal. Limited neural precision is modeled with additive noise (*neural noise*), which sets a constant signal-to-noise ratio (SNR) for individual neurons. To quantify coding fidelity, a *reconstructed signal* is computed from the neural representation with an optimal linear estimator (*decoding*). Note that the decoding aspect of the model is only implicit. The neural portion of the model ends with the neural representation. Finally, various resource constraints can be added further without affecting the reconstruction error, which we will examine later. A formal description of the model is given in Methods.

### Stimulus reconstruction from the neural representation

First, let us observe the advantage of using the proposed model which forms an optimally redundant neural representation. We compare it with a traditional, whitening model which forms a minimally redundant representation. In the whitening model, the encoding filters were chosen to de-convolve and de-correlate the raw sensory signal under the idealized assumption of zero sensory noise [8], [48], [49] (see eq. 8 for the definition; note that whitening is the optimal solution for information maximization over noisy gaussian channels with zero sensory noise). Both models were evaluated with the fidelity of the stimulus reconstruction from the respective neural representations under the same problem settings (i.e., encoding the same ensemble of natural images subject to the same sensory degradation, neural noise, and neural population size). The reconstructed signal was computed with the optimal linear estimator for each model.

**Figure 4:**
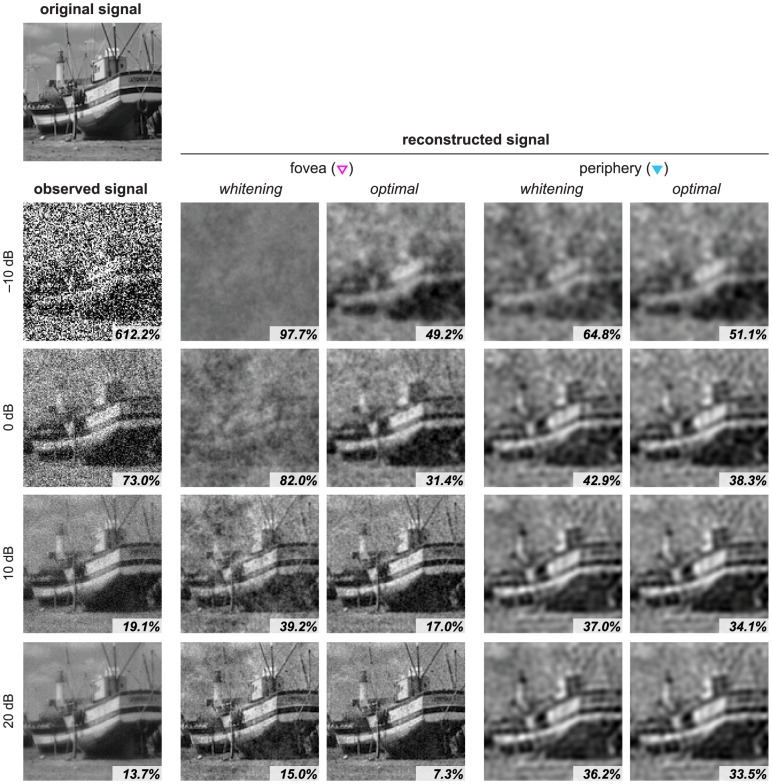
Image reconstruction examples. We compare reconstructions from two different codes: whitening and the proposed, optimal model. The *original signal* (121×121 pixels) is degraded with blur and with different levels of sensory noise (−10 to 20 dB), resulting in the *observed signals*, where the percentage indicates the MSE relative to the original signal. These are encoded under two different cell ratios: 1∶1 (*fovea*) and 16∶1 (*periphery*) for each noise level. The *reconstructed signals* are obtained with the optimal decoding matrices, where the percentage indicates the MSE relative to the original signal, which can also be read out in Figure 5 (labeled by open and closed triangles for the respective eccentricities).

Figure 4 shows reconstruction examples. The sensory noise level was varied from −10 to 20 dB to simulate dark to bright conditions. The neural population size was also varied to illustrate the effect of cell ratio on coding fidelity. Here, we examine two retinal conditions: in the fovea condition, the ratio of pixels (cones) to encoding units (RGCs) was 1∶1; and 16∶1 in the periphery condition. The same optical blur was used for both conditions (30° eccentricity of the human eye [50]) to examine the effect of cell ratio alone. Neural noise was added so that the SNR for each neuron was 10 dB, corresponding to 1.7 bits of information capacity which is consistent with estimates of neural capacity [28].

From these examples, we can make a number of observations. First, the optimal model always (and often significantly) yields better reconstruction than whitening, as should be expected by construction. For example, at the fovea and in the 0 dB sensory noise condition, the reconstructed signal from the whitening model has 82.0% error (in which the boat is barely visible), whereas the proposed model has only 31.4% error. Note that the observed signal initially contains 73.0% error relative to the original signal due to the optical blur and sensory noise. This leads to the second observation that the reconstructed signal can be cleaner than the signal available to a sensory system. It would be useful to recall that our problem is different from a simple, de-noising and de-blurring problem because the reconstruction is also constrained by the limited capacity of the neural representation. Third, the relative advantage of using the optimal code over whitening is higher in the fovea than in the periphery. Under the same, 0 dB condition but in the periphery, the reconstructed error with whitening is 42.9%, whereas the error is 38.3% with the optimal, proposed model – the relative advantage in the periphery is not as significant as in the fovea. Finally, the error is consistently smaller in the fovea than in the periphery with the proposed model, which should be expected because there are more neurons available in the fovea. Interestingly, however, this is not the case with the whitening model when the sensory SNR is low, such as at 0 dB, which we will explain in more detail in the next section.

**Figure 5:**
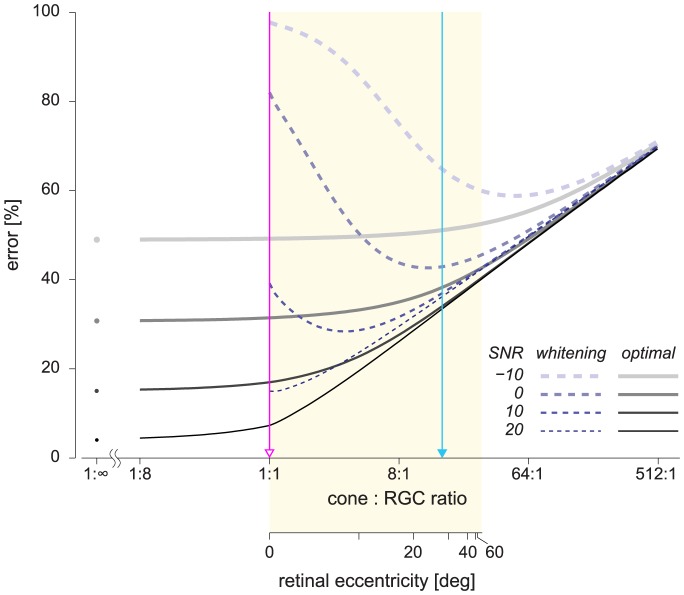
The reconstruction error as a function of neural population size. Two x-axes represent, respectively, the cone: RGC ratio (top) and the corresponding retinal eccentricity in the macaque retina (bottom; see Figure 2). The problem settings are the same as in Figure 4 with extended cell ratios; the common cell ratios (1∶1 and 16∶1) are indicated by the same labels (open and closed triangles, respectively). The signal dimension is 121×121 = 14,641 for all condition; the number of neurons with 16∶1 cell ratio is 915.

The trends of two conditions shown in Figure 4 can be generalized to a continuous range of cell ratios. Figure 5 plots the reconstruction error for the proposed model (solid lines) and whitening model (dashed lines) over a range of population sizes, from large numbers of neurons to very few. The plots show that the relative advantage of the optimal codes is greatest at the 1∶1 cell ratio and diminishes as the cell ratio increases (i.e., the neural population size decreases). Note that the whitening model is not defined for an overcomplete case. In contrast, the proposed model is defined for any cell ratio and is able to reduce the reconstruction error by increasing the population size, up to the limiting case of an infinite population (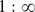 cell ratio). In this limit, there is no loss of information in the neural representation, but there is some error still present inherent to sensory noise and blur [47]. It is also clear that the optimal code yields a large benefit compared to whitening when the level of sensory noise is high. This is also to be expected, because the proposed model takes sensory noise into account while the redundancy reduction model does not. Note that, depending on the sensory SNR, the error reaches an asymptote level with different population sizes. For high SNRs, there is an advantage to having more RGCs relative to cones, whereas for lower SNRs, lower numbers of RGCs are sufficient to encode the available information.

### Mechanisms of optimal representation and reconstruction

**Figure 6:**
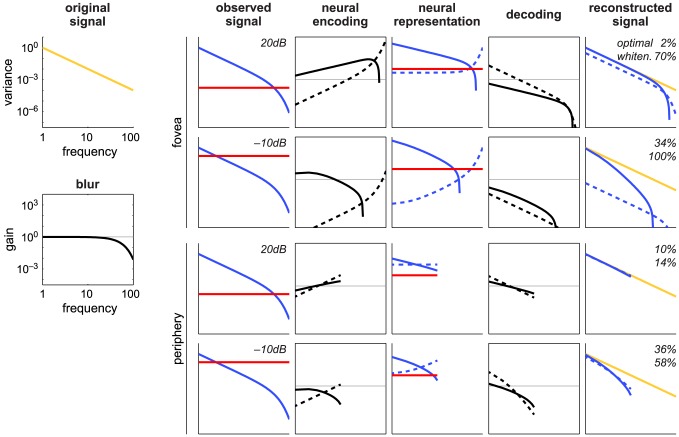
Spectral analysis of the proposed model compared to whitening. Every stage of sensory representations and their transformations are illustrated (cf. Figure 3). The signal is 100-dimensional, and the fovea and periphery conditions differ only in the neural population size (100 and 10, respectively). Each is analyzed under two sensory noise levels (20 and −10 dB). The horizontal axes represent the frequency (or spectrum) of the signal and are common across all plots. The vertical axes of the open plots (e.g., original signal) are common and represent the variance of the indicated sensory representations; those of the box plots (e.g., blur) are also common and represent gain (or modulation) with the indicated transformation, where the thin horizontal line indicates unit gain. The *original signal* (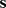, yellow) is assumed to have a 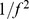 power spectrum where *f* is the frequency of the signal. The *blur* (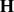, black) is assumed to be low-pass gaussian. The *observed signal* (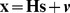) is shown component-wise, i.e., the blurred signal (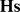, blue) and the sensory noise (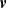, red). The observed signal is transformed by the *neural encoding* (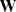, black). Solid and dashed lines indicate the gain as a function of frequency for the proposed and whitening model, respectively (and the same line scheme is used in the other plots). The *neural representation* (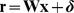) is also shown component-wise, i.e., the encoded signal (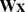, blue) and neural noise (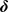, red). The optimal *decoding* transform (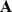, black) is applied to the neural representation to obtain the *reconstructed signal* (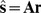; blue), which is superimposed with the original signal (yellow); the percentage shows the MSE of reconstruction. Note all axes are in logarithmic scale. It is useful to recall that transforming a signal with a matrix is multiplicative, but it is simply summation in a logarithmic scale, and thus one can visually compute, for example, the blurred signal as the sum of the original signal and blur curves.

We have seen that the proposed model forms an optimal neural representation for the stimulus reconstruction while whitening fails to do so. To understand how, we can analyze these two models in the spectral domain. The spectral analysis is sufficient to characterize the mathematical mechanisms of both proposed and whitening models that produce different reconstruction errors, because the errors can be expressed solely with the spectral components (see Methods for a formal description). Here, we illustrate the mechanisms using spectral analysis with an idealized model signal (Figure 6).

First, let us examine the fovea (complete code) condition under low sensory noise (20 dB, Figure 6 first row). The observed signal, which consists of the blurred signal (blue curve) and sensory noise (red curve), is transformed by the neural encoding. The spectra of the neural encodings (dashed and solid curves for the proposed and whitening models) represent modulations of the signal in the frequency domain with the respective neural populations. The neural encoding spectrum is a unique characteristic of a population of spatial receptive fields, and we will discuss the characteristics of the spatial form below. In the whitening model, the neural encoding transforms the blurred signal such that the resulting spectrum becomes flat (or white, hence called whitening). In the neural representation, however, the encoded signal (dashed blue curve) is not entirely flat, because it contains the transformed sensory noise in addition to the transformed (whitened) blurred signal. Note that the curve of the whitening neural encoding is by construction vertically symmetric to that of the blurred signal. As a result, whitening amplifies the higher frequency components. This is problematic because the SNR of the observed signal is lower at the higher frequencies. Consequently, in the neural representation, the higher frequencies of the encoded signal have large variances relative to those of neural noise (red curve), but as we have seen, these are the components dominated by the sensory noise. The ideal strategy should be the other way around, which is the one implemented by the proposed, optimal model (see solid blue curve vs. red curve in the neural representation plot).

Specifically, there are two factors underlying the optimal reconstruction in the proposed model. First, highly noise-dominated components at the high frequencies in the observed signal are not encoded at all by the neural encoding, which is truncated roughly where the blurred signal falls below the sensory noise (the exact location of this cut-off frequency was shown to depend on the details of the problem setting [47]). This allows the neural population to allocate its limited representational capacity to high SNR components of the observed signal. This important characteristic is also demonstrated with the two-dimensional toy problem (Text S1 and Figures S1-S5): the optimal receptive fields of two neurons in a population become identical under certain conditions, predicting the most redundant form of code called a *repetitive* code [51]. The second factor is that the optimal model tends to transform the redundant (non-flat) spectrum of the blurred signal into a less redundant (closer to flat) spectrum of the encoded signal, but unlike whitening, this flattening is incomplete (it is exactly halfway when there is no sensory noise, hence called *half-whitening* [47]). With this, the high SNR components of the observed signal have large variances relative to those of neural noise, which is in sharp contrast to whitening.

The basic trends described above also hold with high sensory noise (e.g., −10 dB as in Figure 6 second row) where there are a greater number of low SNR components in the observed signal. The shape of the optimal neural encoding changes accordingly, but that of whitening is identical across different sensory noise levels up to scaling (and hence they are identical up to the vertical translation in the log-log plot). This scaling is a mere reflection of the neural capacity constraint (i.e., the sum of variances in the neural representations is maintained to be a constant while the variance of the observed signal changes with different amounts of sensory noise). With a large amount of sensory noise (−10 dB), nearly 100% of sensory information is lost in the whitening model, because in the neural representation, only high frequency components are greater than neural noise, but they are already corrupted by sensory noise.

Next, we examine the periphery (undercomplete code) condition (Figure 6 bottom two rows). The whitening encoding is exactly the same as in the foveal case except that it has only 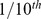 as many components. Notably, this acts as a thresholding mechanism which helps alleviate the aforementioned problem of whitening for the fovea case in which the limited neural capacity was wasted on the noise-dominated, high frequency components. Solely because of this, whitening in the periphery yields an error closer to the optimal value, resulting in (ironically) better reconstruction than whitening in the fovea. This mechanism can be understood more intuitively in the spatial domain. With the unavoidable thresholding effect caused by an undercomplete encoding, the filtering is largely low-pass, which in the spatial domain corresponds to pooling over many pixels. This pooling acts to average out sensory noise and selectively encodes low frequency components. The result is roughly equivalent to encoding only the high SNR components as discussed above. Although these coding mechanisms are common between the proposed and whitening models, it is only the proposed model that adapts its encoding to changes in the sensory noise level (from 20 to −10 dB), leading to a substantial improvement in reconstruction error over whitening (compare errors in the reconstructed signal column).

Finally, this analysis would not be complete without examining an overcomplete case. As observed earlier, the proposed model can have a greater number of encoding units relative to sensory units, and it optimally minimizes the error to the bound set by the sensory degradation (Figure 5). Because the encoding units are noisy, it is beneficial to increase the population size in order to better compensate for the neural noise. The model makes optimal use of added neurons by decreasing the effect of the neural noise in the population, which increases the representational capacity [47]. This highlights an important notion that the neural code is not determined by the ratio of sensory units to encoding units *per se*, but depends on many factors (see Text S1 and Figures S1–S5 for a comprehensive analysis).

### Predicting retinal population coding

**Figure 7:**
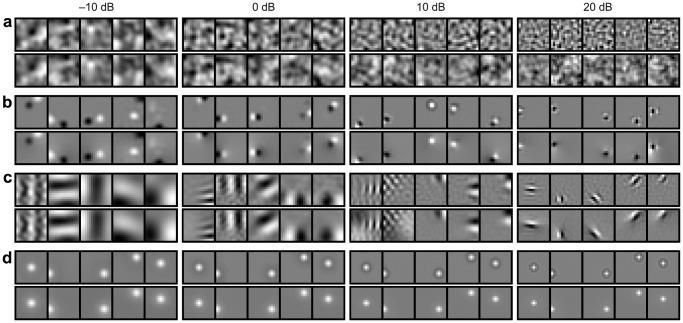
A variety of equally optimal solutions obtained under different resource constraints. Each panel shows a subset of five pairs of neural encoding (top, 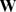) and decoding (bottom, 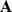) filters in the foveal setting at four sensory SNRs (columns, −10 to 20 dB) in four conditions (rows): (a) No additional constraint (i.e., the base model). (b) Weight sparsity. (c) Response sparsity. (d) Spatial locality. Only the spatial locality constraint yields center-surround receptive fields. See Figure S6 for the resource costs in respective populations. Note that in (d) the center-surround structure is seen only in the filters, which transform the observed signal into the neural code (and hence correspond to receptive fields). The decoding filters have a different, gaussian-like structure. These features are used to optimally reconstruct the original signal from the neural code.

The proposed model predicts how the original signal is optimally encoded in a neural population. The solution is uniquely specified in the spectral domain, however, it does not predict a unique spatial organization of the receptive fields. In other words, there are multiple ways to implement the optimal spectral transform (see Methods for a mathematical explanation of why this arises from the model). Figure 7a shows a subset of optimal encoding (and decoding) filters of the proposed model with no additional constraints. This is a randomly chosen one out of many optimal solutions, and the receptive fields are generally unstructured. Additional constraints are necessary to determine the exact spatial form of the receptive fields.

We investigated three constraints that are relevant to limited biological resources. The first maximized the sparsity of the receptive field weights [41], [43], which could provide an *energy-efficient* implementation of the optimal solution given that synaptic activities are metabolically expensive [52]. This did not, however, yield the types of concentric, center-surround receptive fields found in the retina (Figure 7b).

The second constraint maximized the sparsity of neural responses. This can be justified either by the energy efficiency of the resulting code or from the sparse structure of natural images [7], [8]. This also did not yield concentric center-surround receptive fields, but rather oriented, localized Gabor-like filters which resemble receptive fields found in primary visual cortex (Figure 7c).

Finally, we examined a constraint that maximized the spatial locality of the computation (receptive fields), motivated by the notion that the neural systems generally, and the retina in particular, have limited space and thus should minimize the volume and extent of the neural wiring required to compute the code [39], [42], [44], [53]. With this locality constraint, the model yielded a center-surround receptive field structure, similar to that found in the retina (Figure 7d).

**Figure 8:**
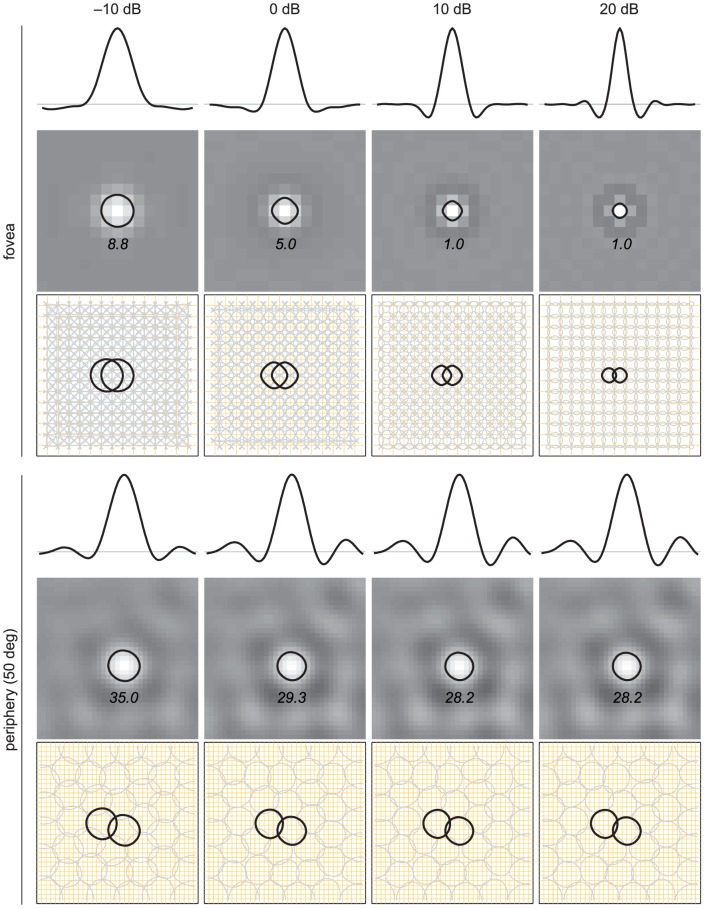
Predicting different retinal light adaptations at different eccentricities. Each panel consists of three plots. *Top*: The (smoothed) cross section of a typical receptive field through the peak. The horizontal line indicates the weight value of zero. *Middle*: The intensity map of the same receptive field. The bright and dark colors indicate positive and negative weight values, respectively, and the medium gray color indicates zero. Superimposed is the outline of the center subregion (the contour defined by the half-height from the peak) along with the average number of pixels (cones photoreceptors) inside the contour. *Bottom*: The half-height contours of the entire neural population which displays their tiling in the visual field. Two neurons are highlighted for clarity (one of which corresponds to the neuron shown above). The pixel lattice is depicted by the orange grid.

With this last constraint, we further examined the details of receptive field structure and organization. Figure 8 shows the prediction at two retinal eccentricities, 0° (fovea) and 50° (periphery). To better model the conditions in the retina, we took into account the optical blur of the human eye [50] and the cell ratio (Figure 2) at the respective eccentricities. As above, we modeled different mean light levels by various sensory SNRs. (Additional information in Methods.)

In the fovea condition, the encoding filters vary from the large, so-called center-only type (−10 dB) to the small, difference-of-gaussian type (20 dB) [15], [54], [55]. This can be expressed in the spectral domain as the transition from low-pass to band-pass filtering (cf. Figure 6). As a result, the overlap of the central region of the receptive fields is very large at the lower SNR, implying that neighboring neurons are transmitting information about a highly overlapped region of pixels at the expense of transmitting independent information. This overlap, however, is optimal for counteracting the high level of sensory noise and encoding the underlying original signal (cf. Figure 4).

In the periphery condition, a similar adaptive change was observed but to a lesser extent. The shape of the receptive field looks similar across all sensory SNRs. More specifically, with the change from 20 to −10 dB, the number of cones inside the central subregion increases only by a factor of 25% in the periphery compared to 780% in the fovea. As was seen in the spectral analysis (Figure 6), the degree of adaptation is limited by the highly convergent cone-to-RGC ratio.

## Discussion

In this article we presented a simple theoretical model of optimal population coding that incorporates several key aspects of sensory systems. The model is analytically well characterized (Figure 6; see also Text S1, Figures S1–S5) and scales to systems with high input dimensionality (Figures 4–5). We found that the optimal code conveys significantly more information about the underlying environmental signal compared to a traditional redundancy reduction model. It has long been argued that some redundancy should be useful [4], [25], [27], [29]–[32], [56]–[59]. Here we provide a simple and quantitative model that optimally incorporates redundancy in a neural population under a wide range of settings. In contrast to earlier studies [24]–[27], [56], [60], the proposed model allows for an arbitrary number of neurons in a population, providing previously unavailable insights and predictions: the degree to and the mechanisms by which the error can be minimized with different input-to-output cell ratios (Figure 6); the conditions in which the redundancy reduction model is near-optimal (Figure 5); the degree of adaptation of receptive fields at different eccentricities to different light levels (Figure 8). We observed that the optimal receptive fields are non-unique, as in other models [8], [25], [59]–[61], and found that the additional constraint of spatial locality of the computation [25], but not previously examined constraints such as sparse weights [41] or sparse responses [7], [8], yielded receptive fields similar to those found in the retina (Figure 7).

A number of other studies have also investigated different optimal coding models that extended the basic idea of redundancy reduction, but with different assumptions and conditions. A commonly assumed objective is *information maximization*, which maximizes the number of discriminable states about the environmental signal in the neural code [6], [25], [27], [56], [57], [59], [62]–[64], whereas the present study assumed *error minimization*, which minimizes the MSE of reconstruction from the neural code [24], [31]. These objectives can be interpreted as different mathematical approaches to the same general goal (some predictions from these different objectives are qualitatively similar [24], [62]; an equivalence can be established between the two under some settings [65]). Recently, Doi et al. [59] showed that the physiologically estimated retinal transform [66] is on average 80% optimal, but note that this model did not uniquely predict concentric center-surround receptive field structures, and that the change of receptive field structure under different conditions (e.g., sensory SNRs and cone-to-RGC ratios) was not examined. Some consequences that arise from the choice of the objective are worth mentioning. One is that de-blurring emerges from error minimization but not from those information maximization models [25], [27], [59], because the error is defined with respect to the original signal prior to blurring. (In [25], [27], [59], the information is defined with respect to the original signal, but it is equivalent to the information about the blurred signal under the model assumptions (eq. 1–2): 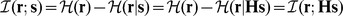, where 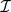 and 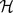 denote the mutual information and the entropy, respectively.) Another is that, in the limit of zero sensory noise, the optimal neural transform for information maximization is whitening (i.e., redundancy is reduced) [25], [27], [59], [64] while that for error minimization is half-whitening (i.e., redundancy is half-preserved) [47].

In many theoretical studies, the input-to-output cell ratio is assumed to be 1∶1, i.e., a complete representation [8], [24], [25], [27]. Although this assumption may be valid in some specific settings such as in the fovea [25], there are many settings in which this assumption is not valid, such as in the periphery (Figure 2). By being able to vary the cell ratio to match the conditions of the system of interest, the proposed model showed that the retinal transform of sensory signals and the resulting redundancy in neural representations vary with the retinal eccentricity. Another common assumption related to the cell ratio is that neural encoding is the inverse of the data generative process [7], [8], where individual neurons are noiseless and represent independent features or intrinsic coordinates of the signal space. In this view, the number of neurons should match the intrinsic dimensionality of the signal. In contrast, in the proposed model the number of neurons may be seen as a parameter for total neural capacity and can be varied independently of the signal's intrinsic dimensionality. Consequently, it is even possible that, while representing an identical signal source, two neurons in the proposed model adaptively change what they represent by changing their receptive fields with different sensory or neural noise levels (Figures S3–S4; notably, two neurons can have identical receptive fields in some extreme cases).

While the current study is based on several simplifying assumptions such as linear neurons with white gaussian neural noise, some recent studies have incorporated more realistic neural properties to investigate the optimality of retinal coding, so it is important to contrast these with the proposed model. Borghuis et al. [57] included instantaneous nonlinearities of neural responses and found that the physiologically observed 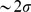 spacing of RGC receptive field arrays [67], [68] is optimal. This is consistent with the prediction of the proposed model under the retinal conditions they studied (i.e., high cone-to-RGC ratios; we estimate the ratio is roughly 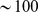, given the reported receptive field size and tiling [57] and the cone density in the guinea pig retina [69]). However, the model presented here predicts that the 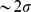 spacing is not optimal in all conditions (Figure 8). Also note that the center-surround structure in their study was assumed, and did not emerge as a result of an optimization as presented here. Pitkow & Meister [64] investigated efficient coding in the retina using a spike count representation and studied the functional role of instantaneous nonlinearity, neither of which was included in this study. Like in the previous study [57], the center-surround receptive fields were measured, not derived. In addition, their analysis assumed zero sensory noise, which as we have shown here can play a significant role in the form of retinal codes. Karklin & Simoncelli [63] proposed an algorithm for optimizing both receptive fields and instantaneous nonlinearities. While they did not assume additional resource constraints or examine different cone-to-RGC ratios systematically, their predictions in certain conditions are consistent with those presented here. Some differences are significant, for example, in their model different types of receptive fields were derived under different sensory and neural SNRs. Further investigations are necessary to bring clarity to these differences. Overall, it is fair to say that there is no model that incorporates all aspects of retinal coding with realistic assumptions, and developing such a model is an open problem for future research. We would point out, however, that there are advantages to simpler models, especially if they can account for important aspects of sensory coding. Some issues that arise with more realistic (and more complex) models are whether they can be analytically characterized, scale to biologically relevant high-dimensional problems, or provide insights beyond simpler models. The proposed model may be seen as a first-order approximation to a complex sensory system and can be used as a *base* model for developing and comparing to models with more realistic properties. Moreover, the optimization of the model is convex, implying that the optimal solution is guaranteed and can be obtained with standard algorithms.

The proposed model made a novel prediction that the change of receptive field structure and organization with different light levels is much greater in the fovea than in the periphery of the macaque midget RGCs (Figure 8). This prediction has not been tested directly because, to the best of our knowledge, all physiological measurements from RGCs with different light levels have carried out either in cat [15], [54], [55] or rabbit [67] retinas, where the reported adaptive changes were marginal. This observation seems to be consistent with our prediction for the periphery, where the cone-to-RGC ratio is high. Note that in the cat retina, the cone-to-RGC ratios (specifically with respect to the most numerous beta RGCs) range from 30 to 200 across eccentricity [70]; in the rabbit retina, we estimate the ratio to be greater than 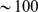, according to the cone density [71], receptive field sizes, and their tiling [67]. If the prediction of larger changes in receptive field structure in fovea conditions (cone-to-RGC ratios near 1∶1) is confirmed by physiological measurements, it would be a strong test of the theory. Note also that some studies have reported larger changes in receptive fields sizes [15], [54], but these were measured between scotopic and photopic conditions. Like previous approaches, here we have only considered cone photoreceptors which implicitly assumes photopic conditions. To include scotopic conditions, one would need to model the rod system [72], [73], which has yet to be incorporated into an efficient coding framework.

The proposed model incorporated a broad range of properties and constraints for sensory systems. It is an abstract model and hence predictions can be made for a wide range of sensory systems by incorporating system-specific conditions. Although we have only modeled conditions for the midget RGCs in the macaque retina, the same framework could be applied to other cell types (e.g., parasol RGCs [68]) or retinas of other species (e.g., cat [15], [54] or human [70]) by incorporating their specific conditions (e.g., cone-to-RGC ratios and optical blur functions). The model can also be applied to other sensory systems, as nothing in the proposed model is specific to the retina. Auditory systems have been approached in the same framework of efficient coding [74]–[77], but the factors introduced in this study have not fully been incorporated into previous models. For example, the cell ratio of sensory units (inner hair cells) to encoding units (auditory nerve fibers) is 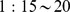 [78], i.e., the neural representation is highly overcomplete, which is very different from the retina (Figure 2). Further, the auditory signal is filtered by the head-related transfer function [79], which could be modeled by the linear distortion in the proposed framework. Olfactory systems have also been studied in an efficient coding framework (e.g., [80], [81]; for reviews, [82]–[84]). It is possible that the optimal redundancy computed with the proposed model may provide insights into olfactory coding beyond decorrelation [81]. Finally, the sensory SNR models the varied intensity of environmental signals relative to the background noise, and the neural SNR models the neural capacity, both of which are broadly relevant. The application of the proposed model to different retinal conditions and other sensory modalities would be a powerful way to investigate common principles of sensory systems.

## Methods

### The problem formulation

We define the linear gaussian model (Figure 3), a functional model of neural responses on which both the proposed and whitening models are constructed. The observed signal 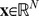 is generated by 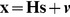
(1)where 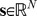 is the original signal, 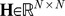 is a linear distortion in the sensing system such as optical blur in vision or the head-related transfer function in audition, and 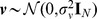 is the sensory noise with variance 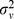, where 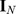 denotes the 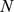-dimensional identity matrix. The covariance of the original signal is defined by 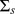. We assume that the original signal is zero mean but need not be gaussian (as in [85]). The sensory SNR is measured in dB, 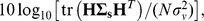 where 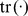 denotes the trace of a matrix. We set the sensory noise variance, 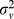, such that the sensory SNR varies from −10 to 20 dB, which covers the physiological range measured in fly photoreceptors (−2.2 to 9.7 dB) [20]. The neural representation 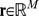 is generated by 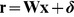
(2)where 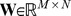 is the encoding matrix whose row vectors are the encoding filters (or linear receptive fields), and 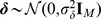 is the neural noise with variance 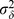. The neural SNR is also measured in dB, 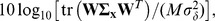 where 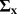 is the covariance of the observed signal, and 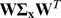 is the covariance of the encoded signal, 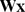. We set the neural SNR to 10 dB so that its information capacity, 1.7 bits, is approximately matched to the values of information transmission estimated in various neural systems (0.6–7.8 bits/spike) [28]. The reconstruction of the original signal from the neural representation is computed by a linear transform 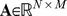


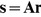
(3)that minimizes the MSE 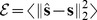
(4)where 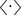 indicates sample average and 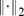 *L* ^2^-norm, given the covariances of signal and noise components in the neural representation (i.e., 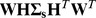 and 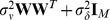, respectively). In other words, the decoding matrix 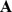 is the Wiener filter which estimates the original signal 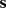 from its degraded version 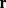 with the linear transform 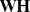 and additive correlated gaussian noise 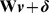 [24], [47]. The proposed, optimal encoding, 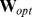, achieves the theoretical limit of the MSE under the linear gaussian model subject to the neural capacity constraint. This constraint can be defined either for the neural population, i.e., with respect to the total variance of neural responses (total power constraint), 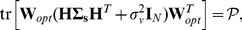
(5)or more strictly for the individual neurons, i.e., with respect to the individual neural variance (the individual power constraint), 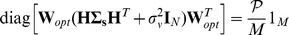
(6)where 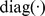 is the diagonal components of a matrix, and 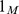 is the *M*-dimensional vector whose elements are all 1. Note eq. 6 implies eq. 5. Importantly, the minimum MSEs under those two conditions are identical [47]. The difference between the two solutions is only in the left orthogonal matrix of the singular value decomposition of the encoding matrix, 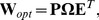
(7)where 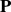 is some *M*-dimensional orthogonal matrix, 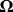 is a unique diagonal matrix whose diagonal elements are the modulation transfer function (or the gain in the spectrum domain) of the encoding, and 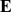 is the eigenvector matrix of the original signal covariance. To summarize, the minimum value of MSE, the coordinates of the encoding (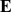), and its power spectrum (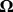) are uniquely determined and in common with the optimization problems with total or individual power constraints. For the derivation of 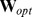, readers should refer to [47].

The whitening matrix, 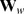, removes all the second-order regularities, both of the signal statistics and of the signal blur [48], and the resulting covariance is the identity matrix with a scaling factor *c*, 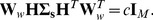
(8)


This scaling is computed such that the neural capacity constraint is satisfied just as in the proposed model (i.e., eq. 5 or 6), namely, 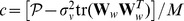. Note that whitening is defined independent of the level of sensory noise 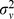 up to this scaling factor, and that the higher is the noise level, the smaller the scaling. This leads to the vertical translation of the whitening spectra at different sensory SNRs (see Figure 6). Finally, whitening for an undercomplete case, 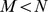, is computed with respect to the first *M* principal components of the original signal as in the prior ICA studies [85].

### Multiplicity of the optimal solution

In general there exist multiple encoding matrices 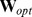 that achieve the optimal MSE. Note the MSE (eq. 4) is invariant with orthogonal matrix 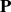 (eq. 7), and so is the total power constraint (eq. 5). Therefore, subject to the total power constraint, 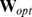 is optimal with any choice of 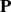. On the other hand, in order to satisfy the individual power constraint (eq. 6), some specific 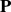 needs to be chosen [47]. The proposed model assumes the individual power constraint so that individual neurons have the same, constant neural precision.

To examine the MSE and the spectrum, there is no need to choose a specific 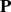 because they are independent of 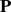. The reconstructed signal depends on the choice of 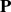 in a weak manner. (The singular value decomposition of the optimal 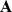 has 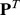 as the right orthogonal matrix, so 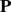 cancels out in the multiplication, 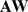. The reconstructed signal is expressed as 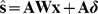, so the choice of 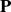 makes a difference only in the second term of the reconstruction, i.e., how the neural noise appears in the reconstruction.) In Figure 4 we used a random orthogonal matrix for 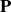 in favor of a large scale image reconstruction; see [46] for reconstructions subject to the individual power constraint but with small image patches.

The receptive field structure depends on the choice of 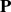, as illustrated in Figure 7. We examined three kinds of additional constraints (on the top of the individual power constraint) to choose 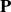: (i) *weight sparsity* measured by the *L* ^1^-norm of the receptive field weights, 
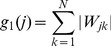
(9)where 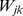 denotes the 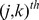 entry of 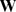; (ii) *response sparsity* measured by the negative log-likelihood with a sparse generalized gaussian distribution, 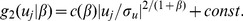
(10)where 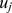 is the 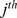 neuron's representation before neural noise is added, 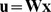, 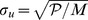 is the standard deviation of the individual neural response, 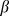 a parameter to define the shape of the distribution (we used 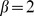), and 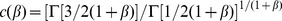 [86]; (iii) *spatial locality* measured by the weighted *L* ^2^-norm of the squared receptive field weights, 
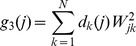
(11)where 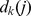 is the weighting (or penalty) defined for each neuron, *j*, by the squared distance between the 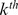 entry and the one with the peak value in 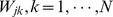.

### An algorithm to derive the solution with an additional constraint

Solutions in Figure 7 which respectively satisfy (a) no additional constraint, (b) weight sparsity, (c) response sparsity, or (d) spatial locality, are derived as follows. Let the individual power constraint of the 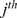 neuron, 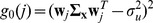
(12)where 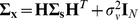 is the covariance of the sensory representation, 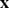.

1. Initialize 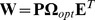 with some *M*-dimensional orthogonal matrix 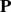.

2. Update 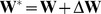 where 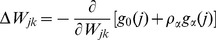
(13)is the gradient of the individual power constraint and the additional constraint, with 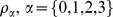 is a parameter which sets the importance of the additional constraint, 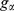 (see eq. 9–11) relative to the individual power constraint, 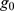. The additional constraint is selected by the index 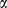, with 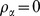 when 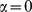 (no additional constraint). Note that 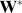 is better in terms of satisfying the constraints than 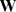, but is no longer guaranteed to be optimal in terms of MSE.

3. Project 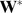 onto the optimal MSE solution manifold subject to the total power constraint, which is parameterized by the *M*-dimensional orthogonal matrix 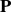. This is solved algebraically by finding the *M* -dimensional orthogonal matrix 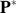 that corresponds to the closest point in the solution manifold in the Euclidean distance, 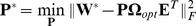
(14)with 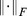 the Frobenius norm [59], [87].

4. Update the solution as 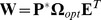.

5. Repeat until 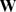 satisfies the convergence criteria for the individual power and additional constraints.

This algorithm is not guaranteed to find a solution, but we observed that it could find solutions with reasonable tolerance for the individual power constraint (i.e., ≤1% of violation; note the total power constraint is exactly satisfied thanks to eq. 14). Figure S6 shows that the additional desired properties (weight sparsity, response sparsity, or spatial locality) were optimized in the respective populations. Finally, we observed that the algorithm is susceptible to local minima.

### An alternative algorithm for the solution with spatial locality

If we could express the desired additional properties of a population of receptive fields in a matrix form, 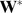, then the optimal solution 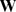 (subject to the total power constraint) closest to 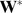 can readily be derived with eq. 14. An important example of this method is with 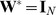 in the complete case. It has been proposed that the retinal transform should minimally change the observed signal to generate the neural representation [88], i.e., 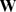 should be as close as possible to the identity, 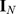. In this case, 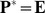, and the encoding matrix is given by 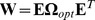. This “symmetric” solution was examined earlier with information maximization [25] and with whitening [88], [89] (which is also called ZCA in the literature [8]).

This algorithm is not limited to the complete case. To derive a spatially localized solution in an undercomplete case, one can set rows of 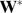 with uniformly tiled gaussian bumps (which may be seen as a generalization of the identity in the undercomplete case). In this study, the locations of the bumps were computed with k-means algorithms with respect to the uniformly distributed samples in the visual field, and the sigma of the gaussians was set by 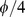 where 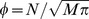 is the radius of ideal (but unrealizable) circles that completely pack the visual field. We examined different values of the sigma from 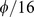 to 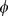, and found that 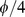 results in the best average locality (eq. 11). The resulting solution is comparable with the one derived with an explicit spatial locality constraint (eq. 11); the spatially localized solutions presented in this article were derived with this alternative algorithm.

### Simulating retinal conditions

There are about twenty types of RGCs in the primate retina which subserve a variety of visual tasks and computations [90]. Here, as in the earlier studies [24], [25], we focus on the computational problem of accurately encoding the image signal with high spatial resolution which is thought to be carried out by the so-called midget type, although the model does not make distinctions among different cell types.

According to the measured cell ratio (Figure 2), we set the number of cone photoreceptors (namely, the number of pixels in the small image region) and that of model RGCs as 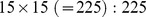 (the ratio is 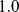) at the fovea, and 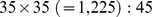 (the ratio is 27.2) at the periphery. The image sizes were chosen to maintain the number of elements in the encoding matrix to be computationally manageable.

### Natural image statistics

Both the proposed and whitening models are adapted to the second-order statistics. Therefore, the solution can be computed only with the covariance matrix of the original signal, 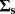. Let 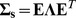 using the eigenvalue decomposition, where 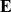 is the eigenvector matrix and 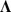 is a diagonal matrix consisting of the eigenvalues (or the power spectrum).

For the image reconstruction of 121×121 pixel images (Figures 4–5), the power spectrum of the original signal (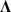) is assumed to be 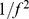 with *f* the spatial frequency. The spectrum at *f* = 0 (i.e., the DC component) is set to zero because the signal is assumed to be zero-mean. The eigenvectors (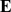) are assumed to be the two-dimensional discrete Fourier basis with the size of 121×121. These two components define a high-dimensional (14,941-dimensional) covariance matrix. Employing this covariance model allowed us to examine image reconstructions in a much larger scale than those in the previous studies (e.g., 8×8 pixel image patches in [31]). In this article we report the MSE in percent error relative to the original signal variance: 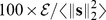.

For the predictions of the retinal code, the signal covariance 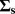 is empirically computed with 507,904 image patches (15×15 or 35×35 pixels) randomly sampled from a calibrated 62 natural image data set [91]. Each image consists of 500×640 pixels with the human L cone spectral sensitivity and the cone nonlinearity. We assigned one pixel to one cone photoreceptor, which corresponds to a sampling density of the human cone photoreceptors of 120 cycle/degree at the fovea and 25 cycle/degree at the periphery (50° eccentricity) [92]. To derive the solution with response sparsity, however, higher-order statistics are required; in this case, we sampled data from the same natural image data set during the optimization.

## Supporting Information

Figure S1 **The optimal solution as a function of signal correlation.**
(EPS)

Figure S2 **The optimal solution in the case of no blur.** These should be compared with the first two cases in Figure S1.
(EPS)

Figure S3 **The optimal solution as a function of sensory SNR.**
(EPS)

Figure S4 **The optimal solution as a function of neural SNR.**
(EPS)

Figure S5 **The optimal solution with different neural population sizes.** *Row 1*: one neuron in the population, or undercomplete case. *Rows 2 & 3*: three neurons in the population, or overcomplete case. These are two different, but equally optimal, solutions. The number labels indicate the corresponding encoding vectors, the axis of neural representations, and the decoding vectors. The two neuron (or complete) case is shown in the middle row of Figure S4.
(EPS)

Figure S6 **Resource costs in equally-optimal solutions.** Resource costs are computed with the solutions presented in Figure 7 with the same labels indicating the type of additional constraints. Each row presents the additional fraction of resource cost relative to the optimized population, i.e., weight sparsity (top, optimized in b), response sparsity (middle, optimized in c), and spatial locality (bottom; optimized in d). Each plot indicates the mean (dot) and the 5^th^ to 95^th^ percentile range (bar), respectively.
(EPS)

Text S1 **Characterization of the optimal solution with a two-dimensional signal.**
(PDF)
