# Supplementary material for: A Simple Model of Optimal Population Coding for Sensory Systems
Source: PLoS Comput Biol. 2014 Aug 14;10(8):e1003761. doi: 10.1371/journal.pcbi.1003761 (PMC4133057; doi:10.1371/journal.pcbi.1003761)
Supplement: Text S1 — Characterization of the optimal solution with a two-dimensional signal. (PDF) [file pcbi.1003761.s007.pdf]

Text S1: Characterization of the optimal solution with a two-dimensional signal

In Figure S1-S5, we present a fuller characterization of the proposed model in a simplified setting, where the original signal is drawn from a two-dimensional gaussian distribution. The goal is to intuitively but fully characterize the base model (i.e., minimizing MSE subject to the individual power constraint without additional resource constraint). There are five factors that determine the solution of this optimization problem: (1) signal statistics (correlation), (2) blur, (3) sensory SNR, (4) neural SNR, and (5) neural population size. We vary one of these at a time and examine the model's behavior.

In these illustrations, an ellipse with principal axes depicts the iso-probability contour of a gaussian probability density at 2σ. The associated dots indicate 100 samples drawn from the density. The color scheme is the same as in the spectral analysis in Figure 6. Each column depicts the following. *Original & blurred signal:* The densities of the original signal, $p(\mathbf{s})$ (yellow) and the blurred signal, $p(\mathbf{Hs})$ (blue), respectively. The pairs of original and blurred samples are connected with lines for clarity. One randomly chosen sample is highlighted with red, which can be traced in the following three plots. The number r^2 indicates the correlation coefficient of the original signal. *Observed signal & neural encoding:* The blurred signal, $p(\mathbf{Hs})$ (blue; same as in the previous plot) and the sensory noise $p(\mathbf{x}|\mathbf{s} = \mathbf{s}^*)$ (red) where * indicates the highlighted sample. Recall the observed signal is $\mathbf{x} = \mathbf{Hs} + \boldsymbol{\nu}$. Also shown are the encoding filters, $\mathbf{W}$ (black). The number indicates the sensory SNR. *Neural representation:* The encoded signal, $p(\mathbf{Wx})$ (blue), the neural noise, $p(\mathbf{r}|\mathbf{x} = \mathbf{x}^*)$ (red), and the total noise components in the neural representation, $p(\mathbf{r}|\mathbf{s} = \mathbf{s}^*)$ (gray), caused by both sensory and neural noise, $\mathbf{W}\boldsymbol{\nu} + \boldsymbol{\delta}$ (because $\mathbf{W}$ is in general not orthogonal, the optimal neural representation contains (anti-)correlated noise) [1]. The number indicates the neural SNR. *Decoding & reconstructed signal:* The decoding, $\mathbf{A}$ (black), the reconstructed signal, $p(\hat{\mathbf{s}})$ (blue), the noise components (or variability) in the reconstructed signal, $p(\hat{\mathbf{s}}|\mathbf{s} = \mathbf{s}^*)$ (gray), and the original signal, $p(\mathbf{s})$ (yellow). The pairs of the reconstructed and the original samples were connected for clarity. The number indicates the MSE of the reconstruction. It would be useful to recall how the signal is reconstructed from the neural representation: $\hat{\mathbf{s}} = \mathbf{Ar} = \sum_{j=1}^{M} r_j \mathbf{a}_j$ where $\mathbf{a}_j$ is the jth column of $\mathbf{A}$, and r_j serves as the coefficient of the vector $\mathbf{a}_j$ to generate $\hat{\mathbf{s}}$.

Signal statistics

In Figure S1, the signal correlation is varied from $r^2 = 0.00$ to 0.70 to 0.95, while all the other conditions are fixed, i.e., the blur is modeled by attenuating the minor component by a constant fraction; sensory and neural noises are added so that the SNRs are both 5 dB; and the number of neurons is two.

We can observe that the two encoding vectors (linear receptive fields) have a wide angle at low correlation. This is partly because the proposed, optimal encoding tries to undo the blurring. The angle gets narrower with higher signal correlation, and eventually, the two vectors collapse, yielding a completely redundant, repetitive code. Note the MSEs get smaller with the more correlated signal. This is because more information about the signal is available to counteract noise as the signal correlation (or regularity) increases.

There are a couple of general points that we can observe from this example. First, it is clear that the optimal encoding and decoding vectors are not similar, $\mathbf{A} \not\propto \mathbf{W}^T$, nor the same, $\mathbf{A} \neq \mathbf{W}^T$ (in contrast to the assumption in the prior studies such as [2]). Second, in some cases, the optimal encoding vectors lean towards the minor axis rather than the major axis (see also Figure S3–S4). This might appear odd on first glance: it seems to make more sense if the encoding vectors were "tuned" to the dimension with greater signal variance. The theoretical analysis clarifies two reasons for this trend. One is de-blurring (i.e., undoing the attenuation along the minor axis). The other is the half-whitening from robust coding (i.e., partially reducing redundancies in the signal). A more formal explanation can be found in [3].

Blur

Figure S2 shows the optimal solution when there is no blur. These two examples are the same as the first two examples of Figure S1 but no blur. The angle between two encoding vectors becomes smaller, because there is no need to undo the blur. Note that the uncorrelated signal ($r^2 = 0.00$) is a special case in which the optimal encoding vectors are given by a pair of orthogonal vectors with an arbitrary orientation (note the vector length needs to be optimized; see [4] for a related analysis).

Sensory SNR

In Figure S3, the sensory SNR is varied. Accordingly, the optimal encoding vectors change dramatically, from strongly enhancing the minor axis (20 dB), to only encoding the major axis (−10 dB). Note that these results correspond to the spectral analysis (Figure 6) and the retinal coding prediction (Figure 8). Namely, encoding vectors at 20 dB lean towards the minor axis of the signal, which in the spectral analysis corresponds to the larger gain in the higher frequencies (recall the signal variances are lower at the higher frequencies, hence corresponding to the minor axis in the 2D example); the encoding vectors get to lean toward the major axis as the signal SNR decreases, which corresponds to the larger gain in the lower frequencies, which in the spatial domain corresponds to the pooling of multiple cone photoreceptors and the highly overlapped receptive field organization.

Neural SNR

In Figure S4, the neural SNR is varied. As in case of varying the sensory SNR, the behavior of the optimal solution changes dramatically with neural SNR, but in a different way. It is common, however, that the two encoding vectors tend to reduce signal's redundancy at the higher SNR, and that the two vectors eventually collapse at the lower SNR.

Neural population size

In Figure S5, the neural population size is varied. If there is only one neuron in the population (row 1), the encoding vector always points to the direction of the major axis of the signal (and so does the decoding vector). If there are three neurons (row 2 & 3), the constellation of encoding vectors becomes irregular, but not completely. This is because $\mathbf{W}_{opt}$ is not fully constrained by the optimization problem: there exist multiple $\mathbf{P}$ in eq. 7 that satisfy the individual power constraint (eq. 6), yielding multiple $\mathbf{W}_{opt}$ (and $\mathbf{A}_{opt}$). The solutions shown in Figure S5 row 2 & 3 are two such examples. Although they look quite different, their encoding (and decoding) spectrum is unique. By imposing an additional constraint such as spatial locality, $\mathbf{P}$ can be uniquely specified, leading to a unique solution of $\mathbf{W}_{opt}$ (and $\mathbf{A}_{opt}$).

References

1. Averbeck BB, Latham PE, Pouget A (2006) Neural correlations, population coding and computation. Nature Reviews Neuroscience 7: 358-366.

2. Vincent BT, Baddeley RJ (2003) Synaptic energy efficiency in retinal processing. Vision Research 43: 1283-1290.

3. Doi E, Lewicki MS (2011) Characterization of minimum error linear coding with sensory and neural noise. Neural Computation 23: 2498–2510.

4. Doi E, Balcan DC, Lewicki MS (2007) Robust coding over noisy overcomplete channels. IEEE Transactions on Image Processing 16: 442-452.
